# Direct comparison of mass cytometry and single-cell RNA sequencing of human peripheral blood mononuclear cells

**DOI:** 10.1038/s41597-024-03399-6

**Published:** 2024-05-30

**Authors:** Emily Y. Su, Kristen Fread, Sarah Goggin, Eli R. Zunder, Patrick Cahan

**Affiliations:** 1 Institute for Cell Engineering, Johns Hopkins School of Medicine, Baltimore, MD USA; 2 Department of Biomedical Engineering, Johns Hopkins School of Medicine, Baltimore, MD USA; 3 Department of Biomedical Engineering, University of Virginia, Charlottesville, VA USA; 4 Department of Molecular Biology and Genetics, Johns Hopkins School of Medicine, Baltimore, MD USA

**Keywords:** Computational biology and bioinformatics, Sequencing, Proteomics

## Abstract

Single-cell methods offer a high-resolution approach for characterizing cell populations. Many studies rely on single-cell transcriptomics to draw conclusions regarding cell state and behavior, with the underlying assumption that transcriptomic readouts largely parallel their protein counterparts and subsequent activity. However, the relationship between transcriptomic and proteomic measurements is imprecise, and thus datasets that probe the extent of their concordance will be useful to refine such conclusions. Additionally, novel single-cell analysis tools often lack appropriate gold standard datasets for the purposes of assessment. Integrative (combining the two data modalities) and predictive (using one modality to improve results from the other) approaches in particular, would benefit from transcriptomic and proteomic data from the same sample of cells. For these reasons, we performed single-cell RNA sequencing, mass cytometry, and flow cytometry on a split-sample of human peripheral blood mononuclear cells. We directly compare the proportions of specific cell types resolved by each technique, and further describe the extent to which protein and mRNA measurements correlate within distinct cell types.

## Background & Summary

Single-cell techniques have revolutionized the resolution at which biology is studied in the lab. Such approaches overcome issues of averaging and subsequent masking of cell-cell heterogeneity experienced by bulk techniques, and thus have become staples in omics research. In particular, single-cell RNA sequencing (scRNA-seq)^1–3^, a high-throughput method to profile transcriptomes, has assisted in cell-type identification^4^, trajectory inference (TI) modeling^5–8^, and gene regulatory network reconstruction^9–11^, to name a few applications. Mass cytometry^12–14^, a high-throughput cytometry method with the ability to simultaneously measure over 40 parameters, has similar applications in cell-type identification^15^, TI modeling^16^, as well as differential signaling and cytokine expression studies^14^.

Transcriptomic data from scRNA-seq is commonly used as a proxy for studying the proteome, as its genomic-scale readout offers a breadth of detail that proteomic approaches are unable to quantify within a single cell. Though broad expression patterns generally associate well with cellular state, the correlation between individual protein expression and corresponding mRNA may be tenuous and even differ amongst proteins or between different cell types^17,18^. These differences can arise from biological sources, including post-transcriptional regulation, or even technical biases, including dropout in scRNA-seq. Thus, improving our understanding of the relationship between measured mRNA and protein content may aid in refining the conclusions drawn from scRNA-seq.

Additionally, integration of scRNA-seq and cytometry is highly enticing as these data modalities are seemingly complementary, with scRNA-seq measuring a large number of features for a relatively low number of observations and vice versa for mass cytometry. Moreover, predictive methods that use one modality to refine the results of the other are equally desirable. Indeed, several such computational approaches have been reported, including COMET^19^, which utilizes scRNA-seq data to infer protein marker panels capable of distinguishing specific cell populations. As these approaches are developed, datasets of both readouts from the same population of cells will be useful as a gold standard for validation.

Here, we performed scRNA-seq, mass cytometry, and flow cytometry on a single, split sample of human peripheral blood mononuclear cells (PBMCs). We further compare the ability of scRNA-seq and mass cytometry in resolving distinct cell types and cell states, describe the extent to which protein and mRNA correlate, and quantify and compare cell-type composition based on data from each technique. Because of the split-sample nature of the work, this is a valuable dataset for the purposes of assessing integrative analyses and resolving measured mRNA-protein relationships. In the development of analysis tools capable of isolating and identifying rare populations, PBMCs are an excellent data source given the existence of well-documented subpopulations and small fraction of certain cell types such as dendritic cells. Such data may be further down-sampled or sub-sampled so as to mimic a sample with even rarer populations. Down-sampling may also enable the exploration of properties of each data modality with respect to accuracy and precision in estimating population structure in the presence of technical factors. Finally, while not presented here, we expect that this dataset can be aggregated with other existing PBMC data in meta-analyses that uncover variation in cell type and states in human PBMCs.

## Methods

Human PBMCs were obtained from a donor, who had provided written informed consent (IRB 15328), at University of Virginia School of Medicine, Heart Center.

### Split-sample preparation for scRNA-seq, CyToF, and flow cytometry

PBMCs were thawed in RPMI 1640 with 5% FBS, and incubated at 37 °C for 1 hr for recovery to ground state. 3 × 10^5^ cells were set aside for scRNA-sequencing. The remaining cells (~7.5 × 10^6^) were divided evenly for mass cytometry and flow cytometry. Cells allocated for scRNA-seq were strained and washed with PBS containing 0.4% BSA. Cell concentration was adjusted to ~500 cells/μL before proceeding with the 10x sequencing protocol.

Next, cells allocated for mass cytometry were fixed. Briefly, cells were incubated with cisplatin (10 µM in PBS) then quenched with cell staining medium (CSM; 0.5% BSA, 0.02% NaN3 in PBS). The cells were strained with a 100 micron nylon strainer before being fixed at room temperature for 10 minutes in 1.6% paraformaldehyde and subsequently stored at −80 °C in CSM. The sample was thawed and stained with metal-conjugated antibodies. Samples are first blocked with 10% donkey serum, stained with surface antibody metal-conjugated antibody cocktail (Table 1), then methanol permeabilized for 10 minutes at 4 °C before being stained for intracellular markers. After staining, samples are incubated with Iridium intercalator for DNA staining overnight at 4 °C before being analyzed on CyTOF mass cytometer (Standard Biotools). Normalization beads containing Lanthanum-139, Praseodymium-141, Terbium-159, Thulium-169, and Lutetium-175 are added to stained samples to perform normalization as previously described^20^. Stained samples and normalization bead mixtures are then filtered through a 40 micron filter and subsequently analyzed across several runs at a rate of ~250 cells per second on the mass cytometer. After measurement, samples are normalized^20^ and de-barcoded^21^ to individual FCS files. FCS files are gated for bead removal, debris clean up, and DNA intercalator.

**Table 1:** CyToF Panel.

Metal Tag and Mass	Specificity
Y89	CD45
In115	CD44
Pr141	CD11b
Nd142	CD79b
Nd144	CD19
Nd145	CD11c
Nd146	IgD
Sm147	CD68
Nd148	CD16
Sm149	CD25
Nd150	CD43
Eu151	CD103
Eu153	CD45RA
Gd155	CD27
Gd156	CD86
Gd157	Nucleoporins
Gd158	CD33
Gd160	CD14
Dy161	Tbet
Dy162	FoxP3
Dy164	CD161
Ho165	CD8a
Er166	CD49a
Er168	CD69
Tm169	CD4
Er170	CD3
Yb171	CD20
Yb172	CD38
Yb173	CD45RO
Yb174	HLADR
Lu175	Perforin
Yb176	CD56

Finally, cells allocated for flow cytometry were blocked with FcBlock (BD, Catalog No. 564219), before they were further divided evenly into six tubes. Primary antibody incubation of each tube was as follows: Tubes 1 and 2, no primary antibody; Tubes 3–6 anti-CD3 (Thermo Fisher, Catalog No. MHCD0300), anti-CD19 (Thermo Fisher, Catalog No. 14-0199-80), anti-CD56 (Thermo Fisher, Catalog No. 14-0567-80), anti-CD14 (Thermo Fisher, Catalog No. 14-0149-80) respectively. Tubes were placed on ice for 30 minutes and washed twice with FACS buffer. Cells were then incubated with secondary antibody (Thermo Fisher, Catalog No. A-11001) for an additional 30 minutes and washed twice before resuspension in FACS buffer. Flow cytometry was carried out on a BD LSR II flow cytometer and analyzed using FlowJo.

### scRNA-seq data processing

**Fig. 1:**
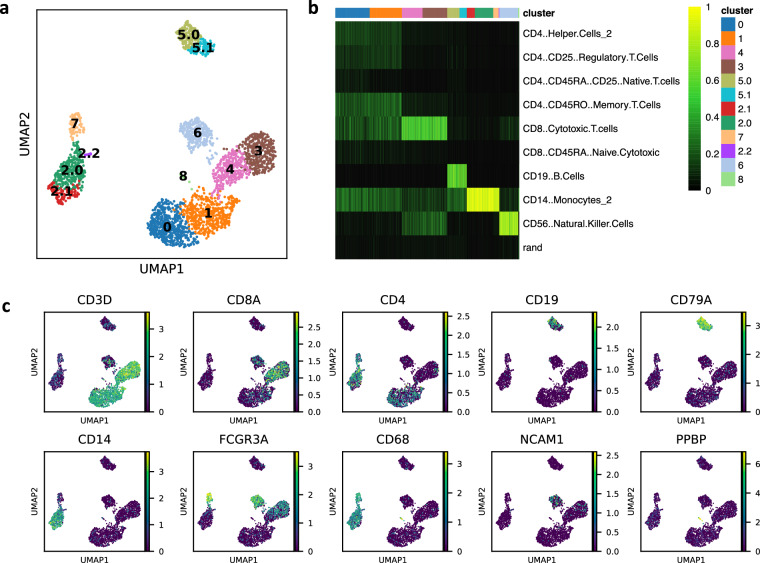
scRNA-seq analysis. (**a**) Clustering result of the scRNA-seq data. (**b**) SingleCellNet classification score heatmap. Reference data was taken from Zheng *et al*. (**c**) Select marker gene expression.

Quality control filtering, normalization, clustering, and differential gene expression analysis was performed using Scanpy^22^ (version 1.8.2). Genes were excluded if they were detected in less than 3 cells; cells were excluded if their mitochondrial gene content exceeded 10% of their total reads or if they had fewer than 200 unique genes in order remove data from any prematurely lysed cells or from ambient RNA. Thresholds were chosen based on manually detecting steep changes in corresponding distributions, aligning with currently accepted practices^22,23^. Of note, varying these thresholds did not significantly change results of downstream analysis (Supplementary Fig. 1, Supplementary Table 1). Filtering resulted in 2653 cells and 15998 genes. The data was then normalized and log transformed and highly variable genes were identified (3004 genes). The data was then scaled and PCA was performed. Cells were clustered using the Leiden algorithm and visualized on a UMAP embedding (Fig. 1a). Further cell type classification was performed via SingleCellNet^24^, using sampled data from Zheng *et al*. as reference data (Fig. 1b). Finally, we annotated cell identity based on these classification results and expression of marker genes (Fig. 1c).

Clusters ‘0’ and ‘1’ expressed CD3D and CD4, and were subsequently annotated as CD4 T cells. Clusters ‘3’ and ‘4’, which expressed CD3D and CD8, classified strongly as CD8 cytotoxic T cells, and were thus annotated as CD8 T cells. Clusters ‘5.0’ and ‘5.1’ expressed CD19, classified strongly as B cells, and were annotated as B cells. Cluster ‘6’, which expressed NCAM1 and KLRD1, classified strongly as natural killer (NK) cells, and were annotated as such. Clusters ‘2.0’ and ‘2.1’ expressed CD14 and CD68, did not express FCGR3A, and classified strongly as monocytes. Both clusters were annotated as CD16- monocytes. Cluster ‘7’ showed markedly lower expression of CD14, high expression of FCGR3A and MS4A7, and classified as monocytes. This cluster was annotated as CD16+ monocyte. Cluster ‘2.2’ did not express CD14 or FCGR3A, but did express CD68, and was annotated as dendritic cells (DC). Finally, the smallest cluster, cluster ‘8’, expressed ‘PPBP’ and is likely a small group of platelets and was annotated as megakaryocyte-lineage (Mk). To note, these annotations can be further divided into finer sub-populations should users choose to refine the clustering, use a different reference dataset for classification, or widen the field of marker genes to analyze.

### CyToF data processing

**Fig. 2:**
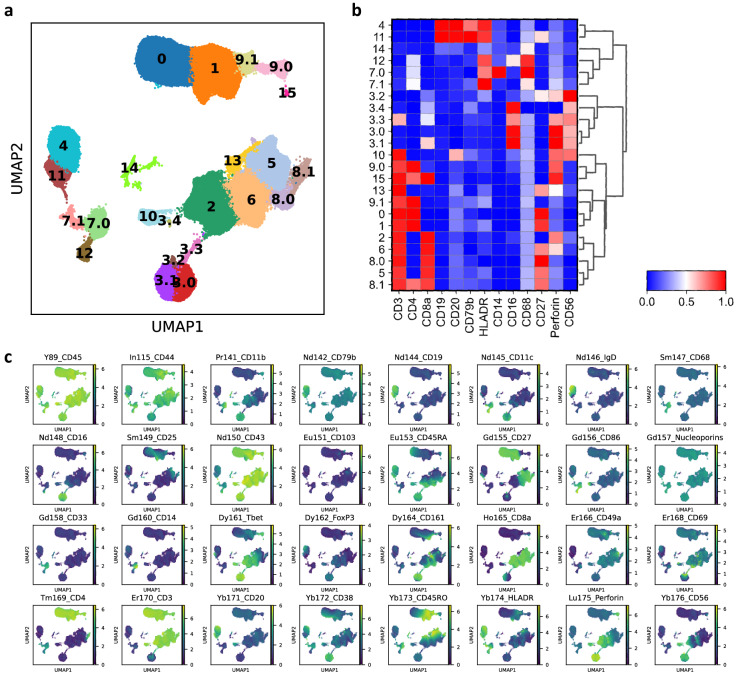
mass cytometry analysis. (**a**) Clustering result of the mass cytometry data. (**b**) Average transformed expression of select markers in each cluster. (**c**) Full mass cytometry panel expression.

Gating to remove debris and subsequent arcsin normalization was done on Cytobank. Leiden clustering and UMAP visualization was performed using Scanpy (Fig. 2a). No other normalization or dimension reduction was performed, and cell annotation was based on marker expression (Fig. 2b,c).

Briefly, clusters ‘0’, ‘1’, ‘9.0’, and ‘9.1’ were CD3+ CD4+, and were annotated as CD4 T cells. Clusters ‘2’, ‘5’, ‘6’, and ‘8.0’, were CD3+ and CD8a+, and were annotated as CD8 T cells. Clusters ‘4’ and ‘11’ were CD19+ CD20+ CD79b+ HLADR+, and were annotated as B cell. Cluster ‘14’ exhibited lower levels of CD19 and CD20, but was also annotated as B cell. Clusters ‘3.0’, ‘3,1’, ‘3.2’, and ‘3.4’ were CD56+ and were labeled as NK cells. Cluster ‘3.3’ were both CD56+ and CD3+, and were labeled as NKT cells. Cluster ‘7.0’ was CD14+ and CD16-, and was annotated as CD16- monocytes. Cluster ‘7.1’ and ‘12’ were HLADR+ and CD68+, and were annotated as DCs. Finally, we found isolated two populations in the CyToF data that we were unable to resolve or detect in the scRNA-seq population. First, Clusters ‘10’ and ‘13’ were CD3+ and both CD4- and CD8a-. We labeled these as double negative T cells (DN T cell). In contrast, cluster ‘15’ was CD3+ CD4+ CD8a+, and was labeled as double positive T cells (DP T cell). To note, based on our Ab panel, users of this dataset can further divide these broad annotations into finer subpopulations.

### Quantifying differences in scRNA-seq and mass cytometry

**Fig. 3:**
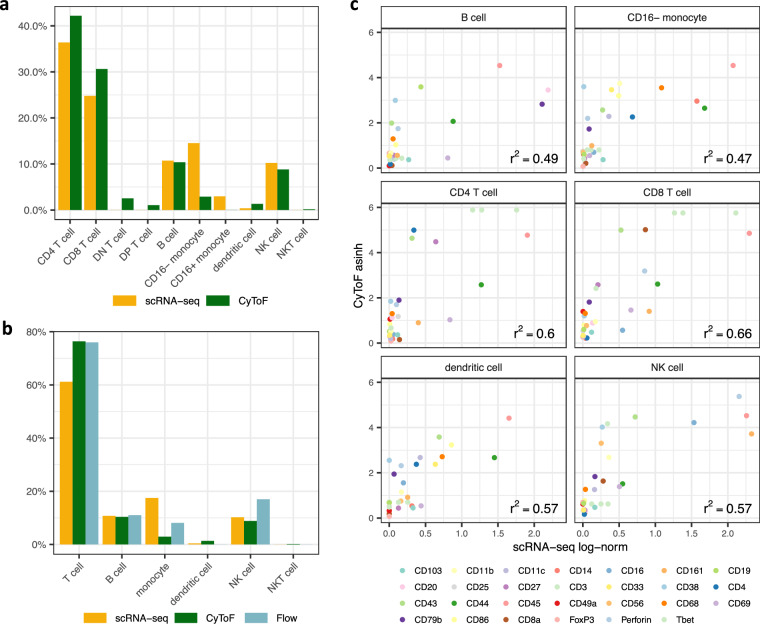
scRNA-seq and mass cytometry comparison. (**a**) Percentage of given cell types in scRNA-seq and mass cytometry data. (**b**) Percentage of given cell types in scRNA-seq, mass cytometry, and flow cytometry data. T cell percentage includes cells annotated as CD4 T, CD8 T, DN T, and DP T. Monocyte percentage includes CD16+ and CD16- monocytes. (**c**) Correlation of CyToF and scRNA-seq measurements in each cell type.

Based on our cluster annotations, we quantified the percentage of each population in the scRNA-seq, mass cytometry, and flow cytometry data (Fig. 3a,b). Importantly, despite the split-sample nature of the three datasets, there was variation in the proportions of specific cell populations. Notably, while mass cytometry and flow cytometry largely agreed in percentage of T cells, scRNA-seq detected a lower percentage of the same population. As described above, this difference was further exacerbated by the DN and DP T cells that were not detected in the scRNA-seq data. In contrast, the scRNA-seq data exhibited a larger proportion of monocytes than both the mass cytometry and flow cytometry data. To note, we did not resolve a CD16+ monocyte population in the mass cytometry data. Finally, while the scRNA-seq and mass cytometry data exhibited a roughly equal proportion of NK cells, the flow cytometry data had a larger percentage of the same population. To note, differences in cell type percentages measured by scRNA-seq and cytometry have been previously reported in bone marrow mononuclear cells (BMMCs)^25^.

To broadly estimate the correlation between scRNA-seq and CyToF measurements, we examined the normalized and log-transformed mass cytometry measurements and compared them to the normalized and log-transformed expression of the corresponding genes in the scRNA-seq data across the different cell types (Fig. 3c). Overall, the correlation between the mass cytometry and scRNA-seq measurements was relatively weak (r^2^ = 0.47–0.66). Taken together, these brief analyses suggest an imprecise concordance between scRNA-seq and mass cytometry measurements. This finding may have broader impacts including suggesting the need for careful consideration in applications such as the identification of rare populations and cell states, which may be obscured using one data modality over another.

## Data Records

The CyToF data^26^ is available at https://flowrepository.org/id/FR-FCM-Z6ZN. The flow cytometry data^27^ is available at https://flowrepository.org/id/FR-FCM-Z6ZX. The scRNA-seq data is available at GEO under accession GSE225431^28^.

## Technical Validation

Common quality control metrics were calculated in Scanpy (including genes per cell, UMI per cell, and percent mitochondrial gene transcripts), and cells were filtered based on these metrics (see Methods). Subsequent singleCellNet classification results of the transcriptomic data largely agreed with the marker gene expression and corresponding cell type annotation (Fig. 1). Additionally, the percentages of each cell type apparent in the scRNA-seq, mass cytometry, and flow cytometry datasets are in line with previously established percentages^3,25,29,30^. Of note, reported PBMC composition proportions vary, likely reflecting both biological and technical variability. For example, one source estimates 70–85% T cells, 5–10% B cells, 5–20% NK cells, 10–20% monocytes, 1-2% dendritic cells, and another measured 49–77% T cells, 6–17% B cells, 7–35% NK cells, 6–12% monocytes, 0.6–1.5% dendritic cells. However, given that our measurements fall within these ranges, we believe this dataset is an accurate representation of human PBMCs. Further, the split sample nature of this dataset ensures that RNA-seq and cytometry measurements correspond to the same sample state.

## Usage Notes

Both raw counts and processed (normalized, log-transformed, with UMAP coordinates) scRNA-seq data are available as H5AD files. Both are stored as AnnData objects. Cluster annotation can be accessed from the ‘.obs’ slot. For the raw dataset, counts data can be found in the ‘.X’ slot. For the processed dataset, the full normalized, log-transformed expression matrix is stored in the ‘.raw’ slot, while the expression matrix subset for highly variable genes is stored in the ‘.X’ slot.

Similarly, the processed CyToF data is available as an H5AD file. Normalized and asinh-transformed data is stored in the ‘.X’ slot. Cluster annotations can be accessed from the ‘.obs’ slot.

### Supplementary information

Supplementary information
