# Supplementary material for: Direct comparison of mass cytometry and single-cell RNA sequencing of human peripheral blood mononuclear cells
Source: Sci Data. 2024 May 30;11:559. doi: 10.1038/s41597-024-03399-6 (PMC11139855; doi:10.1038/s41597-024-03399-6)
Supplement: Supplementary file 1 — Supplementary information [file 41597_2024_3399_MOESM1_ESM.docx]

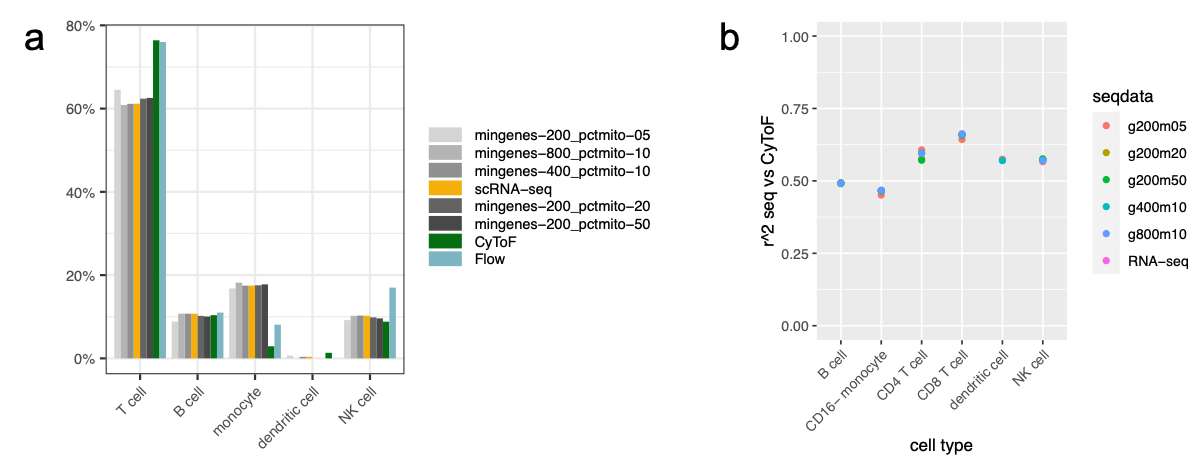


**Supplementary Figure 1. (a)** Percentage of given cell types in scRNA-seq and mass cytometry data. In addition to the original results computed in Figure 3 (yellow, green, blue), results of using different cell filtering thresholds on scRNA-seq data are shown in gray. The scRNA-seq bars are ordered (colored) by number of cells in the filtered dataset. **(b)** Pearson correlation between mass cytometry and scRNA-seq expression by cell type. R-squared values are plotted on the y-axis. Different cell filtering thresholds on scRNA-seq data are colored (example: g200m05 corresponds to  mingenes=200, pct mito=5%).

| Filtering Thresholds | # of Cells in Dataset |
| --- | --- |
| min_genes = 200  pct_mito = 5% | 1522 |
| min_genes = 800  pct_mito = 10% | 2543 |
| min_genes = 400  pct_mito = 10% | 2650 |
| (in the main text)  min_genes = 200  pct_mito = 10% | 2653 |
| min_genes = 200  pct_mito = 20% | 2845 |
| min_genes = 200  pct_mito = 50% | 2899 |
| No filtering | 2909 |

**Supplementary Table 1.** Number of cells in scRNA-seq dataset analyzed given different cell filtering thresholds.

Flow Cytometry Gating Strategies:

| T Cells | CD3+ |
| --- | --- |
| B Cells | CD19+ |
| NK Cells | CD56+ |
| Monocytes | CD14+ |
